# Supplementary material for: Real-Life Comparison of Fosfomycin to Nitrofurantoin for the Treatment of Uncomplicated Lower Urinary Tract Infection in Women
Source: Biomedicines. 2023 Mar 27;11(4):1019. doi: 10.3390/biomedicines11041019 (PMC10136215; doi:10.3390/biomedicines11041019)
Supplement: Supplementary file 1 [file biomedicines-11-01019-s001.zip › biomedicines-2243918-supplementary.pdf]

Supplementary Materials

Real-Life Comparison of Fosfomycin to Nitrofurantoin for the Treatment of Uncomplicated Lower Urinary Tract Infection in Women

Asher Shafrir, Yonatan Oster, Michal Shauly-Aharonov and Jacob Strahilevitz

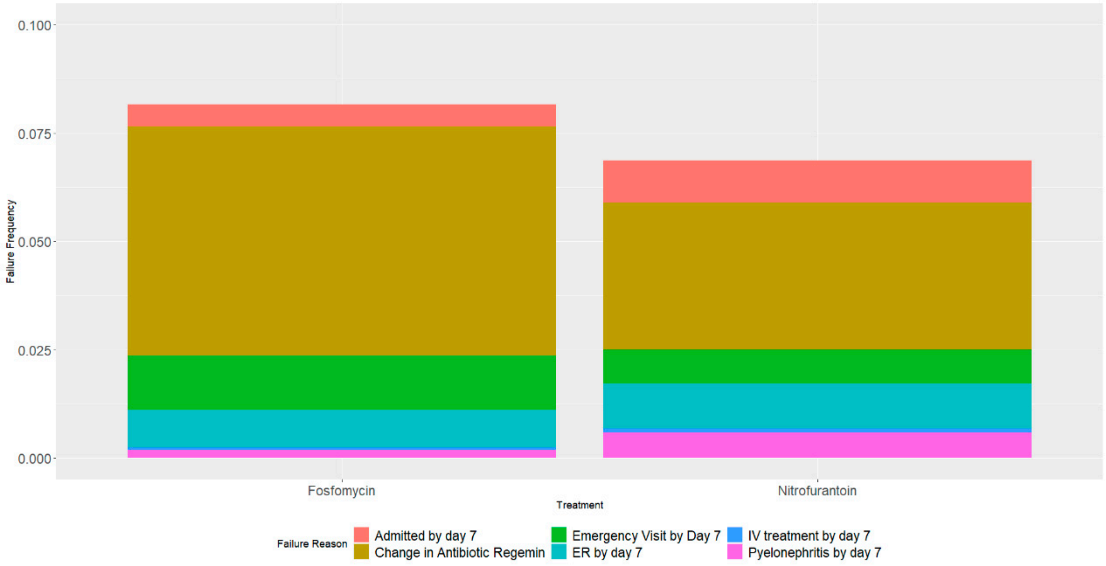

Figure S1. Frequency of Treatment Failure among all patients.

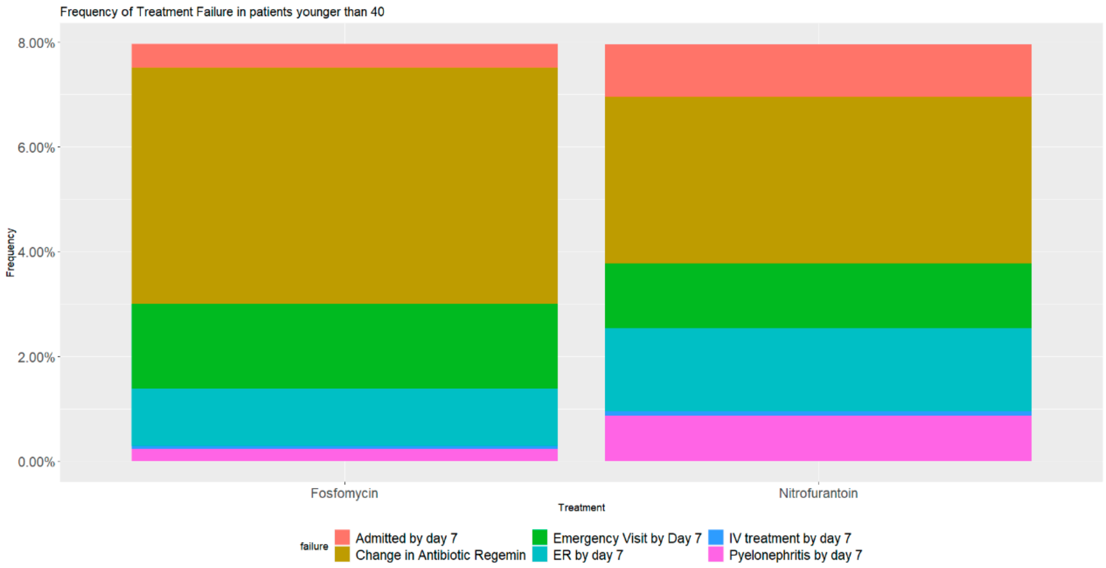

Figure S2. Frequency of Treatment Failure among patients younger than 40 years.

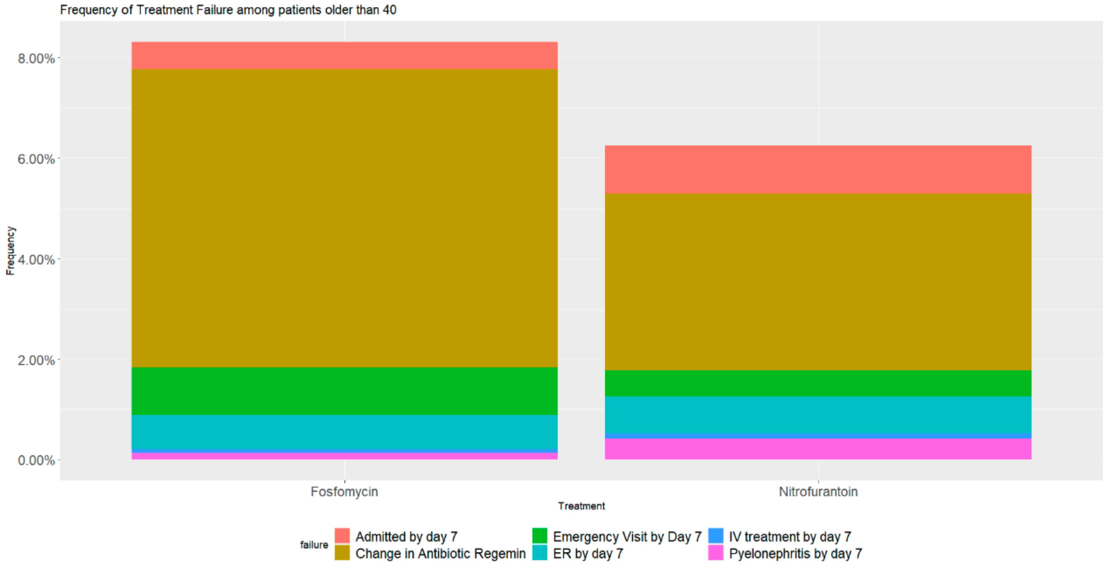

Figure S3. Frequency of Treatment Failure among Patients 40 years or Older.
